# Supplementary material for: Transcriptome Analysis Reveals Vimentin-Induced Disruption of Cell–Cell Associations Augments Breast Cancer Cell Migration
Source: Cells. 2022 Dec 13;11(24):4035. doi: 10.3390/cells11244035 (PMC9776984; doi:10.3390/cells11244035)
Supplement: Supplementary file 1 [file cells-11-04035-s001.zip › DEGs and enrichment analysis.zip!/DEGs and enrichment analysis/WT_VIMvsVector KEGG/UP/WT_VIMvsVector_UP.pathway_map.zip!/WT_VIMvsVector_UP.pathway_map/src/hsa05161.html]

hsa05161


- hsa:332

- Up regulated genes

ENSG00000089685(1.81425481885642)

- hsa:1388

- Up regulated genes

novel.600(2.78845209317737)

- hsa:596

- Up regulated genes

ENSG00000171791(2.03324203294485)

- hsa:1960

- Up regulated genes

ENSG00000179388(2.78490232970451)

- hsa:1870

- Up regulated genes

novel.507(3.55620908288126)
- hsa:1869

- Up regulated genes

ENSG00000101412(1.977544660594)

- hsa:890

- Up regulated genes

ENSG00000145386(1.72013468925064)

- hsa:596

- Up regulated genes

ENSG00000171791(2.03324203294485)

Close
